# Single-Item Patient-Rated Helpfulness and Improvement as an Alternative to Standardized Questionnaires for Establishing Anxiety and Depression Treatment Efficacy

**DOI:** 10.1037/pas0001390

**Published:** 2025-05-15

**Authors:** Patrycja Sewerynek, Caroline Wagner, Thomas McGregor, Megan Skelton, Thalia C. Eley

**Affiliations:** 1 Institute of Psychiatry, Psychology and Neuroscience, King's College London, Denmark Hill, Camberwell, London, UK; 2 National Institute for Health Research (NIHR) Biomedical Research Centre, South London and Maudsley Hospital, London, UK

**Keywords:** Treatment response prediction, Single-item patient ratings, Cognitive behavioural therapy, Outcome measures, Cost-effective assessment

## Abstract

Evidence-based psychological treatments for anxiety and depression are widely used, yet roughly half of those treated do not respond. Treatment response prediction could help to optimise patient outcomes and use of clinical resources. However, existing longitudinal studies with potentially valuable predictors are unlikely to include comprehensive, prospective measures of symptoms throughout therapy. Single-item patient ratings of *helpfulness* and *improvement* are a potentially cost-effective and efficient alternative, but their relationship with typically used change score measures is unknown. Data were analysed from 135 participants (124 female sex; 120 female gender; 127 white) who received Cognitive-Behavioural Therapy. Anxiety symptoms (Generalised Anxiety Disorder-7), depression symptoms (Patient Health Questionnaire-9), and impairment (Work and Social Adjustment Scale) questionnaire scores were obtained before therapy (assessment), before each session, and one-month post-treatment (follow-up). *Helpfulness* (binary) and *improvement* (continuous) ratings were collected at follow-up. Linear regression models assessed the relationship between *helpfulness* and *improvement* ratings and questionnaire change scores from first to last session. Logistic regressions modelled the relationships between single-item measures and NHS Talking Therapies outcomes, derived from questionnaire change scores. *Helpfulness* and *improvement* showed significant associations with questionnaire change scores as well as NHS Talking Therapies outcomes. In a joint model, *improvement* retained significant associations whilst *helpfulness* became non-significant. *Improvement*, and to a lesser extent *helpfulness*, patient ratings may be a cost-effective alternative for establishing treatment efficacy and outcome. The items’ wording and response scales may underlie observed differences. Whilst not equivalent to change score-based measures, they may be adequate for studies requiring large sample sizes.

## Introduction

Anxiety and depression are the most prevalent and debilitating psychiatric disorders worldwide, affecting approximately a third of the population during their lifetime (Ferrari et al., 2013; Kessler et al., 2005). Together, the two disorders are responsible for 7% of years lived with disability (Vos et al., 2017). Despite this, treatment outcomes for anxiety and depression remain suboptimal. Research suggests that current treatments, such as psychological therapy, psychopharmacological drugs, or a combination of both, are effective for only 45% to 65% of individuals with anxiety (Bandelow et al., 2014). Similarly, a meta-analysis of 228 randomised trials comparing psychological therapy for depression against control conditions found that only 41% of patients showed a 50% reduction in depressive symptoms and a mere 30% of patients moved below the clinical cut-off (Cuijpers et al., 2021). The most effective form of psychological therapy for anxiety and depression is considered to be Cognitive-Behavioural Therapy (CBT), yet only around half of individuals respond (Cuijpers et al., 2023; Springer et al., 2018).

The NHS Talking Therapies service (formerly known as Improving Access to Psychological Therapies or IAPT; Clark, 2018) was created to provide evidence-based psychological treatment for anxiety and depression in England. The NHS Talking Therapies manual outlines three definitions of possible treatment outcomes – *Recovery, Reliable Improvement*, and *Reliable Recovery* (NHS, 2021). These outcomes are based on change scores between first and last session from self-report symptom severity questionnaires routinely administered at each session. Specifically, the Generalised Anxiety Disorder-7 (GAD-7; Spitzer et al., 2006) and Patient Health Questionnaire-9 (PHQ-9; Kroenke et al., 2001) scales measuring anxiety and depression symptoms, respectively. Change scores on these two measures from baseline to final therapy appointment are used to determine treatment outcome. Functional impairment (Work and Social Adjustment Scale; WSAS; Mundt et al., 2002) is also regularly assessed during treatment. This is important as impairment is not fully captured by the symptom severity scores and patients consider it one of the most important factors determining their recovery (Zimmerman et al., 2006). Whilst NHS Talking Therapies receives more than 1.8 million referrals annually (NHS, 2022), access to the ‘high-intensity’ treatments is limited due to national cost and shortage of trained healthcare professionals (Blane et al., 2013; Marcus & Olfson, 2010).

One avenue to optimise the use of scarce clinical resources and improve patient outcomes is the development of prediction models for treatment outcomes. These models can then inform personalised treatment approaches. For example, timely identification of treatment non-responders could prevent treatment failure and enable patients to receive an alternative intervention targeted to their personal needs and circumstances (e.g., another treatment, financial or employment support services). This could therefore reduce patient distress and wastage of clinical resources. To train and validate prediction models of who will benefit from treatment for anxiety and depression requires substantial statistical power and thus large sample sizes.

The datasets used to construct these prediction models need to encompass a variety of variables pertinent to psychological treatment response, which clinicians could feasibly gather prior to treatment. One avenue to obtain such data is to use pre-existing large-scale studies, such as the Genetic Links to Anxiety and Depression (GLAD) Study (Davies et al., 2019) or UK Biobank (Sudlow et al., 2015), which contain extensive data that could be relevant to predicting treatment response. However, pre-existing datasets are unlikely to contain detailed, prospectively collected, pre-/post treatment outcome scores such as those found in NHS Talking Therapies datasets. Collecting detailed treatment outcome information is time and cost intensive and, in cases where linking to NHS Talking Therapies data is not possible, alternatives must be explored. To support research efforts in treatment outcome prediction, it is necessary to determine time and cost-effective methods of assessing treatment efficacy. Even when repeated outcome information is available, the common practice of focusing on change scores can fail to sufficiently capture residual symptoms or impairment that are particularly impactful on the patient (Sacchetti et al. 2015; Lutz et al., 2013).

A potential solution to these issues is to use simple retrospective measures of treatment response. For example, after a course of therapy, patients could answer a single question about whether they found their treatment *helpful* or whether their symptoms and day-to-day functioning *improved* after their treatment course. Single-item self-report measures of treatment outcome would therefore be useful for retrospectively capturing treatment information in studies without medical records, or for intervention studies with limited resources for continual assessments. Single self-reported items have successfully been used to vastly increase sample sizes, and thus statistical power, within genetic studies of anxiety and depression (Davies et al., 2022, Howard et al., 2019).

Retrospective reports may prove to be a good marker of treatment efficacy and outcome. However, previous research suggests that prospective measures (comparison of pre- and post-treatment) and retrospective measures (post-treatment) of surgical treatment outcomes are not equivalent and that retrospective reports produce higher estimates of treatment benefits (Aseltine et al., 1995). Although change score measures are also created from self-reported items, these measures are often thought of as more objective than retrospective single items. This is because they are based on up-to-date appraisals of diagnostic symptomatology, as opposed to retrospective assessment that is a potentially biased evaluation of past experiences. This implies that retrospective reports and prospectively assessed change scores may not be equivalent and the nature of their relationship with pathophysiology may differ. In sum, it is crucial to understand the extent to which existing clinical outcome measures relate to patient-rated *helpfulness* and *improvement* to determine the feasibility of using the latter in research on psychological treatment efficacy.

In this study, we investigated the relationships between pre-/post-treatment GAD-7, PHQ-9, and WSAS change scores with single-item patient reports of *helpfulness* and *improvement* collected one month after treatment. To further assess the utility of *helpfulness* and *improvement* in inferring anxiety and depression treatment efficacy we explored their associations with three binary change score-based NHS Talking Therapies treatment outcomes. We hypothesised that increased symptom and impairment severity (as measured using GAD-7, PHQ-9, and WSAS change scores, respectively) would be significantly negatively associated with retrospectively reported *helpfulness* and *improvement*. We further hypothesised that *helpfulness* and *improvement* would be significantly positively associated with each of the binary NHS Talking Therapies treatment outcomes. We tested these hypotheses using linear and logistic regression models.

## Methods

### Transparency and Openness

All analyses were conducted in R version 4.3.1 (R Core Team, 2023). This investigation stemmed from an undergraduate research project and the present analyses were not pre-registered. See Table S2 in the supplementary materials to access the link to the code used. The anonymised data and research materials used in this study are available upon request.

### Participants

In this investigation, secondary analyses of the data from the Predicting Outcomes in Response to Therapy (PORT; McGregor et al., 2025) were conducted. Participants in the PORT study were invited from a broader study, the Genetic Links to Anxiety and Depression (GLAD; Davies et al., 2019) Study, a re-contactable sample of around 40,000 volunteers with lifetime experience of clinical anxiety and/or depression. To participate in PORT, individuals had to meet screening criteria assessed through an online survey and pass safety and suitability checks conducted by phone. These are described in Figure S1 of the supplementary materials, alongside the inclusion and exclusion criteria relevant to this study.

The final sample consisted of 135 young adults (aged: 19–32 years, *M* = 27.0, *SD* = 3.1) who attended between 2 and 15 CBT treatments (*M* = 6.5, *SD* = 3.3). Most participants were diagnosed with either generalised anxiety disorder (44.4%) or a depressive episode (28.1%). The remainder were diagnosed with other types of emotional disorders (see Table S1 of the supplementary materials). Participants mainly identified as female gender, with only nine identifying as male, and six as non-binary. With respect to sex assigned at birth, there were 124 females, and 11 males. Their ethnic origin was mainly white, with 127 participants identifying as such, seven as mixed, and one identifying as other. Most participants were in full-time employment (61.5%), with the rest classified as students (16.3%), working part-time (9.6%), self-employed (5.2%), or unemployed/other (7.4%).

The PORT study received ethical approval from the Ethics Committee at King’s College London (reference: HR/DP-20/21-21094). Participants provided written informed consent before taking part in the study.

### Procedure

A baseline survey was completed by participants approximately two weeks prior to treatment starting (M = 12.2, SD = 9.2 days), which included the GAD-7, PHQ-9, and WSAS measures. Eligible participants were referred to the external therapy provider to receive a course of CBT. Roughly one month after their last treatment session (M = 44.07, SD = 21.23 days), participants completed a follow-up survey. The follow-up survey assessed patient experiences of treatment *helpfulness* and *improvement*. The survey was not administered at the last treatment session as it was a feature of the study rather than an NHS measure administered during treatment. Baseline and follow-up surveys were administered via REDCap (Harris et al., 2019). They received a monetary reimbursement as a shopping voucher of £20 for completing the baseline assessment survey, and a further £10 for completing therapy and the follow-up survey. Explanatory and outcome variables were derived from measures collected at baseline assessment, before each CBT treatment session, and at follow-up.

#### Treatment

Participants received CBT via a commercial platform developed by ieso for use within NHS Talking Therapies for anxiety and depression. CBT was conducted over a confidential internet instant-messaging platform with a therapist accredited by the British Association for Behavioural and Cognitive Psychotherapies. As in all NHS Talking Therapies services, participants received a ‘problem descriptor’ (referred to as ‘diagnosis’), based on International Classification of Diseases-10 codes. Although not formal diagnoses, these descriptors inform the selection of the disorder-specific CBT protocol to be used in treatment. The protocols are recommended treatments by the National Institute for Health and Care Excellence (NICE, 2011).

### Measures

#### Explanatory Variables

##### Helpfulness

A single-item measure of patient-rated treatment *helpfulness* was collected at follow-up, through the question “Did you find your sessions with ieso helpful?”. Participants could respond by selecting either “yes” or “no”, coded 1 and 0, respectively.

##### Improvement

Participants completed a single-item measure to indicate their level of symptom improvement at follow-up: “How much did your symptoms and day-to-day functioning improve after your sessions with ieso?”. The response options were: “much better”, “little better”, “no change”, “little worse”, “much worse”, respectively coded as 4-0. We operationalised this variable as continuous in keeping with guidance suggesting this is appropriate with five or more categories (Norman, 2010; Sullivan & Artino, 2013).

#### Outcome Variables

##### Generalised Anxiety Disorder 7-Item Scale

The GAD-7 is a seven-item self-report measure of generalised anxiety symptoms (Spitzer et al., 2006). Participants reported how often they had been bothered by certain symptoms (e.g., “feeling nervous, anxious or on edge”) over the past two weeks. Response options were “not at all”, “several days”, “more than half of the days”, or “nearly every day” scored 0-3, respectively. Item scores were summed to give a total ranging from 0-21. The NHS Talking Therapies cut-off for clinical anxiety is ≥ 8 (NHS England, 2021). The GAD-7 has previously shown excellent internal consistency (Cronbach’s alpha = .92) (Spitzer et al., 2006). This was reflected in the internal consistency (Cronbach’s alpha = .91) of the GLAD study from which this investigation stemmed (Davies et al., 2019).

##### Patient Health Questionnaire 9-Item Scale

The PHQ-9 is a nine-item self-report measure of depression symptoms (Kroenke et al., 2001). Participants were asked to report how often they had been bothered by, for example, “feeling down, depressed, or hopeless”, over the past two weeks. The response option wording and scoring were the same as for the GAD-7. Summed item scores provided a total ranging from 0-27. The NHS Talking Therapies case threshold is ≥10 (NHS England, 2021). The PHQ-9 has previously demonstrated excellent internal consistency (Cronbach’s alpha = .89; Kroenke et al., 2001). In the wider GLAD study, Cronbach’s alpha was found to equal .90, indicating good internal consistency (Davies et al., 2019).

##### Work and Social Adjustment Scale

Participants’ level of functional impairment was operationalised using the self-report WSAS (Mundt et al., 2002). The WSAS assesses functional impairment across five domains: close relationships, ability to work, home management, social leisure activities, and private leisure activities. Items are worded after the following pattern “Because of my [disorder], my [life domain] is impaired”. Participants scored each of the five items on a nine-point Likert scale ranging from zero (“no impairment”) to eight (“very severe impairment”). Item scores were summed to obtain a total score between 0 and 40. A score above 20 is considered to indicate a severe level of functional impairment. Cronbach’s alpha for this scale has previously shown between acceptable and excellent values across a range of different disorders, including obsessive compulsive disorder and depression (*α* = .70-.94; Mundt et al., 2002).

### Variable creation

#### Change scores

Change scores (Delta, Δ) from the ieso intake assessment to the last treatment session were calculated for each participant to quantify change in symptoms (anxiety, ΔGAD-7; depression, ΔPHQ-9) and impairment (ΔWSAS). These were derived for each scale by subtracting the score obtained at the ieso intake assessment (~ 1 week before the first CBT session) from the scores collected at the final treatment appointment for that individual. A negative change score therefore indicates improvement, while a positive value indicates deterioration. When the assessment score was missing (true for two participants for GAD-7, three for PHQ-9 and three for WSAS), we instead used the score from the first treatment session. As participants completed the questionnaires prior to each appointment, scores from this first session would not have been biased by treatment effects. There are different schools of thought with regards to the use of change scores (Laird, 2020). We favoured their use over other approaches such as linear mixed or multilevel models because they align with the established assessment of treatment outcomes in clinical practice and enable comparability with the large body of existing research using change scores. This avoids methodological heterogeneity in line with recommendations for treatment outcome research (Loerinc et al., 2015). However, due to concerns surrounding the use of change scores, we additionally ran linear mixed models in a supplementary analysis.

#### NHS Talking Therapies Treatment Outcomes

Three standard NHS Talking Therapies variables were derived from the symptom measure scores: Recovery, Reliable Improvement, and Reliable Recovery (NHS England, 2021; see Table 1 for full definitions). These are used by the NHS to monitor the effectiveness of treatments. Due to the high comorbidity between emotional disorders, it is recommended that treatment outcomes should be based on a joint consideration of GAD-7 and PHQ-9 measures (NHS England, 2014). Treatment outcome can therefore be determined in terms of an individual’s overall internalising symptomology, rather than a single psychological condition. For our analyses, each treatment outcome was a separate outcome variable (1 = “yes”; 0 = “no”; *NA*).

### Data Pre-Processing and Statistical Analyses

Correlations were computed between *helpfulness* and *improvement* and each of the questionnaire change scores. These were Point-Biserial correlations (Kornbrot, 2014) for *helpfulness*, and Pearson’s correlations for *improvement*.

Overall, nine linear and nine logistic multivariable regressions were computed and relevant assumptions were tested (see Table S3 of the supplementary materials). Across all models, we adjusted for number of treatment sessions, gender, and age as evidence suggests these are related to psychological treatment outcomes (Eskildsen et al., 2010; Nilsen et al., 2013; LeBlanc & Ritchie, 2001), but as they were not our focus of interest their coefficients were not interpreted. None of the explanatory variables or covariates were excessively skewed (skewness score < |1|). We standardised continuous covariates (number of treatment sessions and age). Variables based on GAD-7, PHQ-9, and WSAS questionnaires were not standardised to preserve the scales of measurement of interest to our research.

#### Linear and Logistic Regressions

Nine linear regression models were performed. The outcomes were ΔGAD-7, ΔPHQ-9, and ΔWSAS. For each outcome, we tested a univariable model with the explanatory binary variable *helpfulness*, another univariable model with the continuous variable *improvement* and a third multivariable model with these two explanatory variables together. As well as inspecting the beta coefficients and significance values, we also calculated the adjusted R^2^ to determine the proportion of variance explained by each model.

Nine logistic regression models were fitted, with each NHS Talking Therapies treatment outcome (*Recovery, Reliable Improvement, and Reliable Recovery*) as a separate outcome variable. Again, three models were performed per outcome. The explanatory variables were the same as for the linear regressions (a univariable model for *helpfulness*, a univariable model for *improvement*, and a multivariable model with both *helpfulness* and *improvement*). The probability of falling into a positive category of *Recovery, Reliable Improvement, or Reliable Recovery* was compared between the explanatory variables to enhance the understanding of the relationship between these measures. Probabilities were calculated using the formula: $probability\;=\frac{\exp(\log\;odds\;)}{1+\exp(\log\;odds\;)}$.

Finally, for descriptive purposes, the proportion of agreement was calculated between the categories of patient-rated treatment *helpfulness* and *improvement* (here treated as categorical) and each of Recovery, *Reliable Improvement*, and *Reliable Recovery*.

#### Linear Mixed Models

To address concerns associated with the use of change scores, we conducted complementary analyses using linear mixed models. For each standardised questionnaire (GAD-7, PHQ-9, and WSAS) we estimated a linear mixed model with the questionnaire score at the final treatment session as the outcome variable, and the baseline questionnaire score, helpfulness, improvement, age, gender and number of treatment sessions as explanatory variables. To account for individual variability, we included a random intercept for each participant.

#### Corrections for Multiple Testing

Corrections for multiple testing were applied using the Holm-Bonferroni adjustment (Holm, 1979), with a threshold of .05, adjusting for the expected proportion of false positives among the significant results. In total, four Holm-Bonferroni corrections were run, each containing six *p*-values: for linear regressions, *p*-values from the six models with a single predictor variable were grouped, and *p*-values from the three models with two predictor variables were grouped, and the same approach was taken for the logistic regressions. Adjusted *p*-values are reported in Table S6 of the supplementary materials.

## Results

Change scores (Δ) on the clinical questionnaires (GAD-7, PHQ-9, and WSAS) across positive categories of NHS Talking Therapies outcomes Recovery, Reliable Improvement and Reliable Recovery were consistently largest for the GAD-7, followed by the WSAS (see Table S7, supplementary materials). The least change was seen in PHQ-9 scores. Independent samples t-tests showed that the change scores for patients that met positive criteria for each of the NHS Talking Therapies treatment outcomes categories were significantly greater (*p* < 0.001) than those that did not (see Table S8, supplementary materials). The point-biserial correlation between helpfulness and improvement was moderate to strong (rpb = .64). The GAD-7 and PHQ-9 correlated moderately (r = .54) at the first treatment session, and very strongly at the last treatment session (r = .81).

### Associations between patient-rated helpfulness and improvement, and clinical questionnaire change scores

The correlations between *helpfulness* and *improvement*, respectively, with change scores were -.30 and -.45 for ΔGAD-7, -.20 and -.42 for ΔPHQ-9, -.28 and -.44 for ΔWSAS. Therefore, the magnitude of the correlations was greater for improvement, compared to helpfulness. A bar plot visualising the mean (standard deviation) change in each clinical questionnaire score (GAD-7, PHQ9 and WSAS) and the five different categories of *improvement* can be found in Figure S2. The observed pattern suggests a linear association between *improvement* and clinical questionnaire change scores. The unequal distribution of participants across the different categories of improvement, with solely two participants in the ‘much worse’ category, should be considered when evaluating the results.

**Figure 1:**
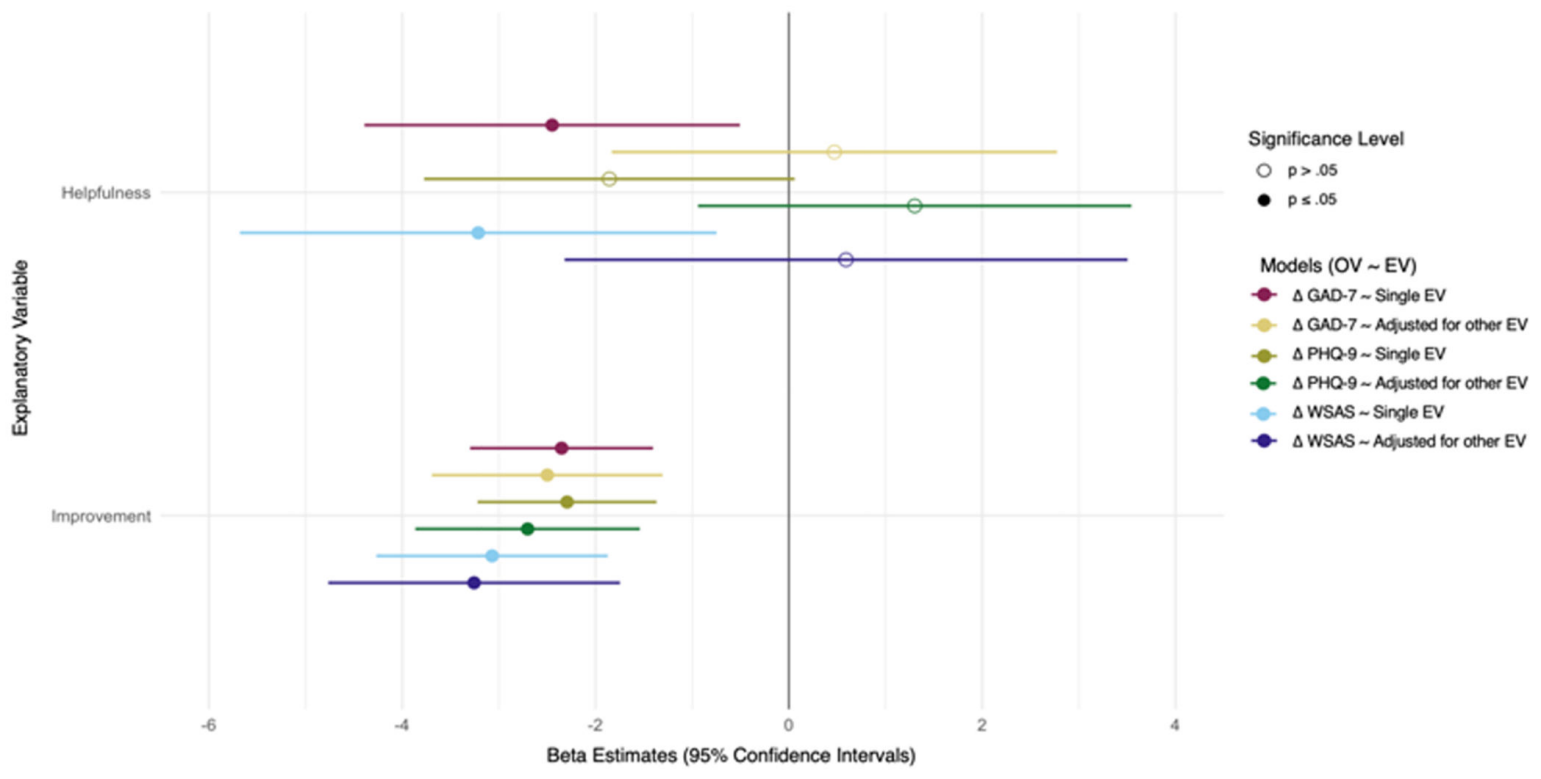
Linear regressions of pre-/post-treatment change scores and patient rated outcomes of helpfulness, improvement, and a multivariable model of both helpfulness and improvement. *Note*. GAD-7 = Generalised Anxiety Disorder 7-item Scale; PHQ-9 = Patient Health Questionnaire 9-Item Scale; WSAS = Work and Social Adjustment Scale; Δ = change scores; OV = Observed Variable; EV = Explanatory Variable.

Forest plots visualising the results for the univariable and multivariable linear regression models between pre-/post-treatment change scores and the single-item measures are presented in Figure 1. Across all models, patient-rated *improvement* was significantly associated with change scores (*β*s -2.30 – -3.07, *p* < .001). *Helpfulness* was significantly associated with change scores for anxiety (*β* = -2.43, *p* = 0.01) and impairment (*β* = -3.22, *p* = 0.01), but not depression (*β* = 1.87, *p* = 0.06). See Supplementary S6 for full regression results. The magnitude of the statistically significant effect sizes cannot be interpreted according to Cohen (2013) since they are dependent on the outcome measure. Nevertheless, we observed that the magnitude of the effect was similar between helpfulness and improvement in univariable models, and became larger for improvement compared to helpfulness in multivariable models.

### Associations between patient-rated helpfulness and improvement, and NHS Talking Therapies treatment outcomes

**Figure 2:**
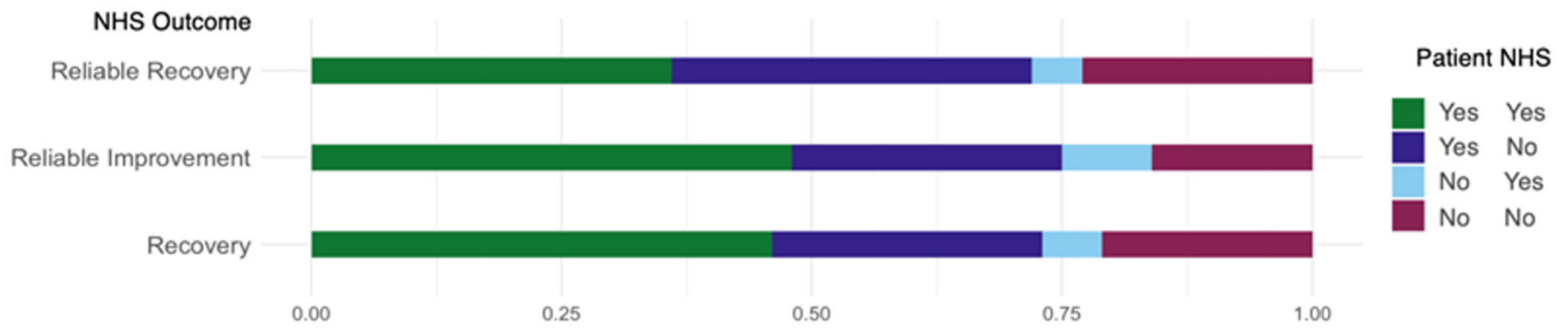
Proportion of agreement between patient-rated helpfulness and NHS Talking Therapies treatment outcomes. *Note*. For example, in the recovery category, 48% did not meet recovery (NHS = No; dark blue and burgundy). 27% did not meet recovery and reported that they found treatment helpful (NHS = No & Patient = Yes), and 21% did not meet recovery and did not find treatment helpful (NHS = No & Patient = No).

**Figure 3:**
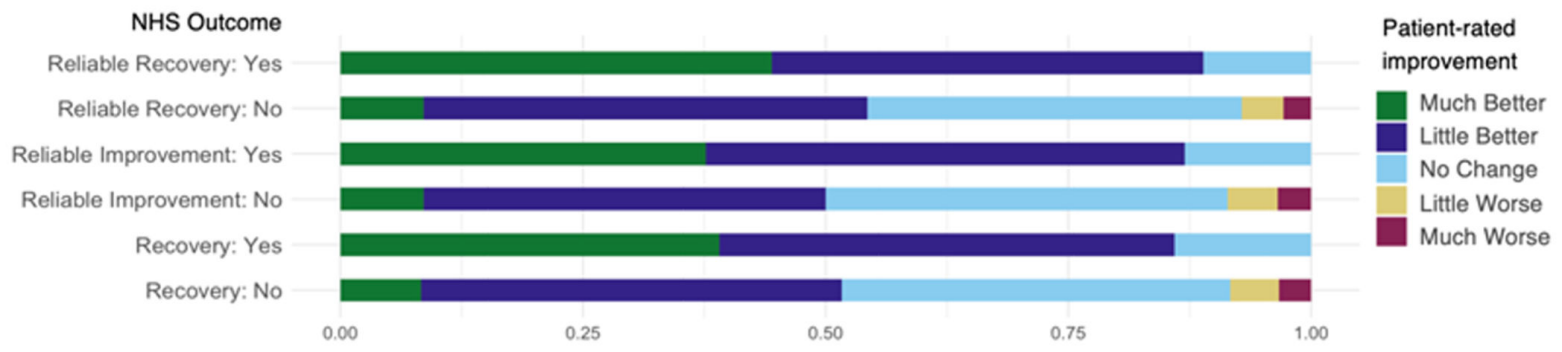
Proportion of agreement between patient-rated improvement NHS Talking Therapies treatment outcomes.

Figures 2 and 3 present the agreement between each category of NHS Talking Therapies treatment outcomes and patient-rated *helpfulness* and *improvement* respectively. A notable proportion of participants who did not meet any of the NHS Talking Therapies treatment outcomes (Recovery, Reliable Improvement, Reliable Recovery) reported treatment as *helpful* (27%, 27%, 36%, respectively) or in terms of improvement were either *much better* (8%, 9%, 9%) or *little better* (43%, 41%, 46%). However, among those who did have positive outcomes for the NHS Talking Therapies categories named Recovery, Reliable Improvement, or Reliable Recovery, only 6%, 9%, and 5% reported treatment as not *helpful*, respectively. None of the individuals who indicated on the improvement scale that their symptoms got worse fell into the positive outcome categories.

**Figure 4:**
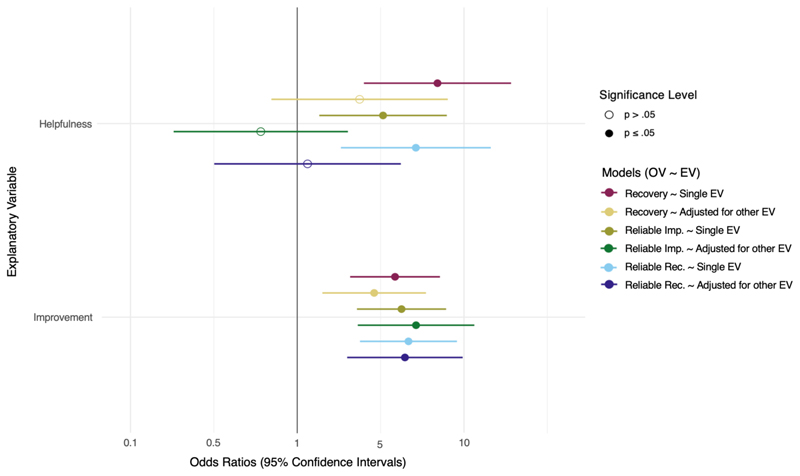
Logistic regressions of NHS Talking Therapy treatment outcomes and patient rated outcomes of helpfulness, improvement, and a multivariable model of both helpfulness and improvement. ***Note***. EV = Explanatory Variable

The forest plot in Figure 4 visualises the results from the logistic regressions. They show the respective associations between each NHS Talking Therapy treatment outcome in a univariable model with *helpfulness*, a univariable model with *improvement* and a multivariable model with both *helpfulness* and *improvement*. Results from the univariable models showed that both *helpfulness* and *improvement* were significantly associated with Recovery, Reliable Improvement, and Reliable Recovery (*ORs* 3.29 - 6.92, *p* ≤ .01). The magnitude of these odds ratios indicates a strong to very strong effect (Rosenthal, 1996). Across the three multivariable models, only improvement was significantly associated with NHS Talking Therapies treatment outcomes (*ORs* 2.89 – 5.16, *p* ≤ .01), suggesting a medium to large effect size (Rosenthal, 1996). See supplementary Table S6 for full regression results. The probabilities of meeting the criteria of NHS Talking Therapies treatment outcomes (based on logarithmic odds derived from regression models) by the categories of *helpfulness* and *improvement* are depicted in Figure S3 of the supplementary materials. To further investigate the robustness of the reported results post-hoc, we repeated both the linear and logistic regression analyses without holding number of treatment sessions, gender and age constant. The univariable regression between ΔPHQ-9 and helpfulness became statistically significant (*p* = .04). Effect sizes remained similar. No other notable differences were observed, and therefore, for brevity, the full results are not reported.

The conclusions from the above analyses using change scores did not substantially differ from the results obtained through linear mixed models. Results from the linear mixed models suggested that helpfulness is not significantly associated with treatment outcome for any of the three measures (*βs* -.97 - .20, *p* = .34 - .98). Improvement, on the other hand, was significantly associated with treatment outcomes. For those associations, beta-values of -2.31, -2.89, and -3.51 (GAD-7, PHQ-9, WSAS; all *p*-values <.001) indicated that a one-unit higher score on improvement was associated with a 2-3 point decrease on each outcome measure. Please find the full results of the linear mixed models in Table S4 of the supplementary materials. Analyses of variance (ANOVAs) were run to evaluate whether the variance components of the random effects were greater than zero by comparing the mixed models to the equivalent linear models. Lower information criteria values indicated that the linear models of final score were a slightly better fit of the data, although not significantly (see Table S5 of the supplementary materials for the full results).

## Discussion

This study investigated whether retrospective patient-rated measures of *improvement* and *helpfulness* are associated with change scores in anxiety symptoms, depression symptoms, and impairment from pre-to post-treatment. We further sought to understand whether these explanatory variables were associated with a patient’s classification into NHS Talking Therapy treatment outcomes (Recovery, Reliable Recovery, and Reliable Improvement). As we hypothesised, *helpfulness* and *improvement* were significantly associated with both change scores and NHS Talking Therapies treatment outcomes when examined individually. However, when considered together only the effect of *improvement* remained significant. The effect sizes of significant effects ranged from medium to very large, further confirming our hypotheses.

The significant associations and large effect sizes between the measure of *improvement* in particular, but also *helpfulness*, with established measures of treatment outcomes suggest that they may be an effective alternative for assessing treatment efficacy. These associations could mean that retrospective reports of improvement are reflective of an individual’s self-reported prospective symptoms across time. In practice, these single-item self-report measures could benefit both academic research and clinicians as they are less resource-intensive to collect and could be applied retrospectively within existing longitudinal studies. This is likely to be particularly beneficial for research requiring large sample sizes, such as the development of prediction models of treatment outcome. On the other hand, the magnitude of the correlations between *helpfulness* and *improvement* with change scores were weak to moderate. Together, this suggests that while measures of *helpfulness* and *improvement* may not be equivalent to the assessment of treatment outcomes using change scores, they are still good indicators thereof. It highlights that, to a certain extent, a trade-off has to be made between the comprehensiveness and the cost-/time-efficacy of measurements assessing treatment outcomes. In addition, an almost identical pattern of results was found when computing these models without the covariates, contributing to the potential for replicability of the observed effect. Notably, these self-report measures appear to indicate greater perceived patient benefit than that identified by change scores or the NHS Talking Therapies treatment outcomes.

In addition to differences in the wording of the items, the greater variability within the response options of the continuous measure of *improvement* compared with the binary variable of *helpfulness* may underlie its stronger associations with change score treatment outcomes. With each increasing level of improvement, the mean change in clinical questionnaire scores decreased (indicating a positive change in patient symptoms). This indicates that, where single item self-report measures of treatment outcomes are used, they should offer a range of response options rather than only “yes” or “no”. At the same time, it should be noted that we are not able to determine whether the difference in results is due to the greater variability in the improvement variable or its content. A small variance for helpfulness is revealed in the small covariances and is consistent with the lower effect sizes of the bivariate correlations for helpfulness. The correlation between *helpfulness* and *improvement* suggested that they are related but distinct concepts.

Findings also suggested that the functional impairment change score (ΔWSAS) had the strongest associations with helpfulness and improvement compared to the symptom change scores (ΔGAD-7 and ΔPHQ-9). This reveals that these measures could capture more than just symptoms, but also general functioning, which is key to patient’s perceptions of recovery (Zimmerman et al., 2006). This reveals a limitation in the wording of the *improvement* self-report question because it asks about both improvement in day-to-day functioning and symptom improvement. Future studies should ask patients separately about functioning and symptom improvement such that researchers can differentiate the two concepts in their analyses.

An alternative interpretation of these findings is that the measures of *helpfulness* and *improvement* might capture features that are not directly related to the diagnostic criteria of a patient’s condition. This has been proposed to account for differences in genetic studies using single items of self-reported diagnosis compared with full diagnostic criteria (Cai et al., 2020; Schwabe et al., 2019). The measures of *helpfulness* and *improvement* might reflect symptom severity for internalising disorders (which anxiety and depression fall into; Khan et al., 2005) more broadly, rather than the severity of symptoms that are specific to the questionnaires used in the present study. For example, while the GAD-7 is sensitive to a range of anxiety disorders, it mainly captures generalised anxiety disorder, rather than symptoms of other disorders treated in NHS Talking Therapies, including obsessive-compulsive disorder and post-traumatic stress disorder (Kroenke et al., 2007). To further illustrate, a patient with major depressive disorder may show an overall improved symptomatology related to their condition while still having suicidal thoughts. This patient would have greater change scores but might report feeling as though treatment was not helpful due to the persistence of their suicidal thoughts. Future research is necessary to investigate the reasons underlying the differences observed in the present study.

### Constraints on Generality and Limitations

The participants recruited in this study were volunteers with a lifetime experience of clinical anxiety and/or depression who participated in a broader study and agreed to be recontacted. A sampling bias may have occurred because participants were recruited through a social media campaign targeted to a demographic between the ages of 16-30, resulting in a sample consisting mainly of young adult females. While on the one hand this highlights a limited sample diversity and thereby generalisability of the results, it also represents a demographic where these mental illnesses are particularly pronounced, and appear to be increasing (Dykxhoorn et al., 2023). The high proportion of females in this study (92%), was similar to the proportion of participants in the wider PORT study (90%). Although this gender imbalance is more pronounced in our sample, it reflects a documented disparity in access to psychological treatment services (Pattyn et al., 2015). In 2021, 68% of individuals accessing psychological therapies through the NHS were female (NHS, 2021). In addition, participants were recruited from a sample where 94.8% of participants identified as white (Davies et al., 2019). This proportion was reflected in the demographics of the present study, where 94.1 % described their ethnic origin as white, 5.2% as mixed, and 0.74% as ‘other’. This proportion is higher than within the population of England and Wales (82%; Census, 2022) and should therefore be considered when evaluating the generalisability of the findings. A sampling bias in the recruitment methods may be an underlying cause for these exacerbated differences. For example, the participant sample was initially recruited through social media campaigns, and females may be more likely to interact with mental health content online compared to males. To better understand the generalisability of these findings, future studies should investigate the use of single-item measures of *helpfulness* and *improvement* to establish treatment efficacy with probabilistic sampling methods.

These findings should be considered in light of several limitations. First, it is possible that the higher estimates of treatment benefit from the measures of *helpfulness* and *improvement* compared to the change score-based measures were biased by the patient’s symptom and impairment severity at the time of completion (i.e. one month after treatment). If the participants’ symptom severity and impairment biased their responses, it would suggest that individuals had improved at follow-up compared with the end of treatment. Although generally symptoms are very similar or slightly increased following treatment (Lutz et al., 2009; Yoshinaga et al., 2019), this could occur if individuals continued to practice what they learnt in treatment, or due to improvements in other external circumstances. This highlights how an individual’s experiences in the intervening period between treatment completion and their retrospective self-report of helpfulness or improvement could influence their perception of treatment outcome.

Second, all measures were based on self-reports and were not compared to any formal post-treatment diagnoses or observer-rated outcomes. Although the change score measures and the NHS Talking Therapies treatment outcomes were based on standardised questionnaires for anxiety and depression symptoms (Kroenke et al., 2001, Spitzer et al., 2006), these measures are still self-reported and cannot be thought of as strictly objective. A final and important limitation relates to the use of change scores. Their use may have introduced a distortion or bias in the observed associations within this study. This is associated with issues of endogeneity and having the single-item variables interact with time as well as using them as predictors of the outcome. Despite the availability of more sophisticated statistical methods, we chose change scores to align with the methods currently used in clinical practice to assess treatment outcomes. This approach facilitates the direct comparison between our results and the current clinical practice, as well as its associated body of research. In doing so, we address concerns (e.g. Loerinc et al., 2015) that a heterogeneity in measures and methods used to assess treatment outcomes often restricts cross-study comparisons and implementation of findings. To assuage these concerns, we implemented a series of linear mixed models, for which the results substantively aligned with findings from the models using change scores. This suggests that the reported findings are independent of the modelling decision and supports the robustness of the reported findings.

To conclude, using single-item patient-rated measures of *improvement*, and to a lesser extent *helpfulness*, may be an effective time-efficient approach to assessing treatment efficacy.

## Supplementary Material

supplementary materials

## Figures and Tables

**Table 1:** Definitions and conditional assignment criteria of NHS Talking Therapies treatment outcomes – Recovery, Reliable Improvement, Reliable Recovery (NHS England, 2021).

Treatment outcome		Definitions
Recovery		“Yes” for individuals who were above the clinical cut-off for either or both of the GAD-7 (≥ 8) and PHQ-9 (≥ 10) at assessment and at the end of treatment scored below the cut-off for BOTH GAD-7 and PHQ-9. Otherwise, “no”. If patients did not score above either symptom measure at assessment, they were scored as missing for this outcome.
Reliable Improvement		“Yes” for patients who had either: (1) decrease (improvement) of ≥ 4 points on GAD-7 scale and of ≥ 6 points on PHQ-9, or (2) decrease of ≥ 4 points on GAD-7 and on PHQ-9 change (increase/decrease) of < 6 points, or (3) decrease of ≥ 6 points on PHQ-9 and on GAD-7 change (increase/decrease) of < 4 points. “No” for patients who did not meet any of the above criteria.
Reliable Recovery		“Yes” for individuals who met the criteria for both Recovery and Reliable Improvement. If missing for Recovery, scored as missing. Otherwise, “no”.

*Note*. Δ = change; GAD-7 = Generalised Anxiety Disorder 7-item Scale; PHQ-9 = Patient Health Questionnaire 9-Item Scale. Treatment outcomes are based on the NHS Talking Therapies definitions.

## Public Significance Statement

This study suggests that single-item patient-ratings of *improvement* and *helpfulness*, could potentially be used as a cost-effective and efficient alternative to change-score based measures when establishing the outcome of psychological treatment for anxiety and depression. These measures could be particularly valuable for analyses of treatment outcome requiring large sample sizes, where it is unfeasible to collect the prospective data needed for change scores.
