# Supplementary material for: Single-Item Patient-Rated Helpfulness and Improvement as an Alternative to Standardized Questionnaires for Establishing Anxiety and Depression Treatment Efficacy
Source: Psychol Assess. Author manuscript; Available in PMC 2026 Jan 6. (PMC7618587; doi:10.1037/pas0001390)
Supplement: supplementary materials [file EMS211757-supplement-supplementary_materials.docx]

**Supplementary Materials**

**Table S1**

*Prevalence of participant diagnoses* based on International Classification of Diseases-10 codes *attributed to patients by the NHS Talking Therapy services.*

| Diagnosis Name | Number of diagnosed participants | Percentage of participants |
| --- | --- | --- |
| Generalised anxiety disorder | 60 | 44.4 |
| Depressive episode | 38 | 28.1 |
| Panic disorder episodic paroxymal anxiety | 9 | 6.7 |
| Social phobias | 8 | 5.9 |
| Agoraphobia with or without panic disorder | * | * |
| Recurrent depressive disorder | * | * |
| Obsessive compulsive disorder | * | * |
| Post-traumatic stress disorder | * | * |
| Somatoform disorders | * | * |
| Specific isolated phobias | * | * |
| Adjustment disorders | * | * |

*** Count masked as ≤ 5 observations**

**Figure S1**

*Relevant Exclusion-Inclusion Criteria, with associated sample sizes displayed on the left.*

Number of Participants:

**Table S2**

*Access to the code used in this investigation for each part of the data analyses.*

| Description | Link |
| --- | --- |
| Github Repository | https://github.com/50280/KURF |

**Table S3**

| Assumption | Tests Conducted |  |
| --- | --- | --- |
| Linearity of the data | Visual assessment of scatterplots for each explanatory variable and covariate and outcome variable (or logit of outcome variable for logistic regressions). |  |
|  |  |  |
|  |  |  |
|  |  |  |
| Homogeneity of variance | Levene's test. |  |
|  |  |  |
|  |  |  |
| Independence | Observations came from separate individuals. |  |
|  |  |  |
|  |  |  |
| Multicollinearity | Correlation matrices and the magnitude of the Variance Inflation Factor. The highest observed magnitude was 2, below the guideline threshold of 5 (Shrestha, 2020). |  |
|  |  |  |
|  |  |  |
|  |  |  |
| Extreme Outliers | Plotting Cook’s distance and calculating the standard deviation for the points visually identified as outliers. No point exceeded 2 standard deviations from the mean. |  |
|  |  |  |

*How assumptions were tested for linear and logistic regressions.*

**Figure S2**

*Association between the mean (standard deviation) GAD-7, PHQ-9 and WSAS change scores, for each category of improvement.* **
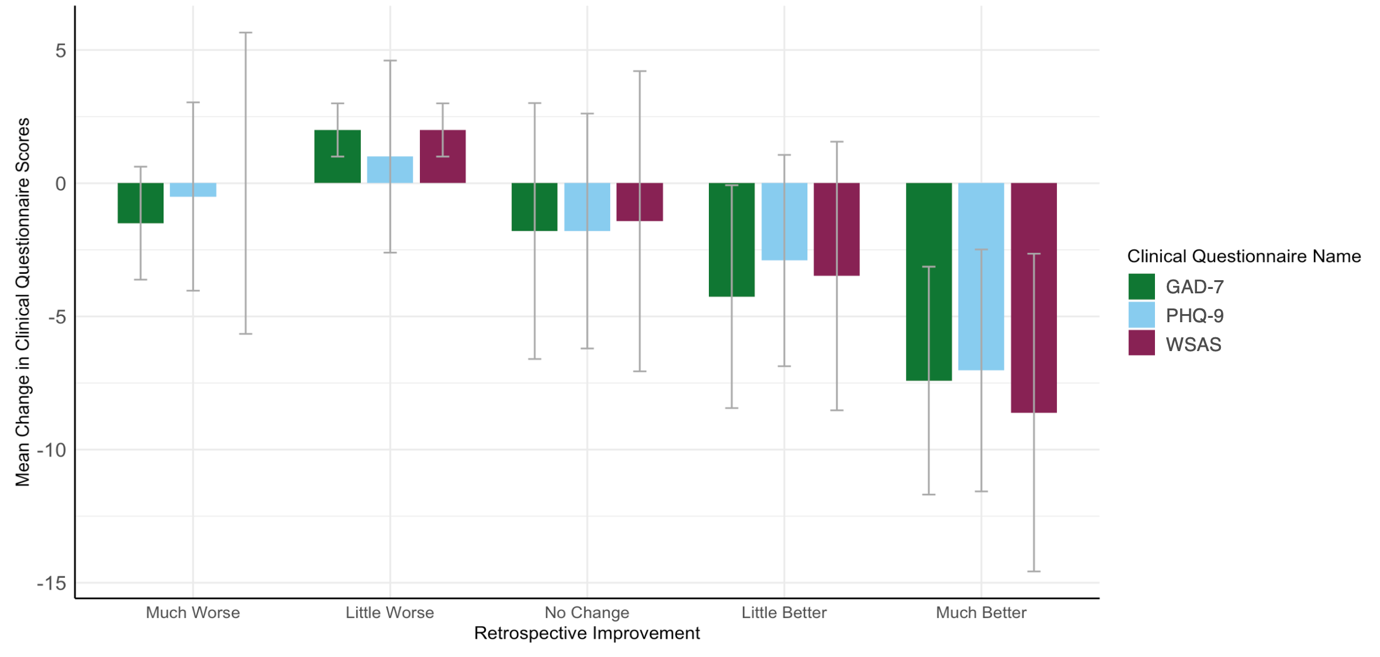
***Note.* From ‘Much Worse’ to ‘Much Better’ the respective number of participants in each category were ≤5, ≤5, 34, 62, 34.

**Table S4.**

Outputs and model summaries for linear mixed models that include baseline symptoms as explanatory variables, a random intercept for each participant, and final session score as the outcome.

| Outcome variable | Predictor | *β* | *SE* | *df* | *t*-value | *p*-value | AIC | Random Effects |
| --- | --- | --- | --- | --- | --- | --- | --- | --- |
| GAD-7 | Intercept | 11.20 | 1.98 | 128 | 5.66 | <.001 | 786.82 | 3.43 |
|  | First treatment | 0.45 | 0.08 | 128 | 6.00 | <.001 |  | Residual variance: 1.29 |
|  | Helpfulness | -0.97 | 1.00 | 128 | -0.97 | 0.34 |  |  |
|  | Improvement | -2.31 | 0.51 | 128 | -4.55 | <.001 |  |  |
|  |  |  |  |  |  |  |  |  |
| PHQ-9 | Intercept | 10.16 | 2.09 | 128 | 4.86 | <.001 | 741.91 | 3.37 |
|  | First treatment | 0.50 | 0.07 | 128 | 7.11 | <.001 |  | Residual variance: 1.26 |
|  | Helpfulness | 0.20 | 0.97 | 128 | 0.21 | 0.84 |  |  |
|  | Improvement | -2.89 | 0.50 | 128 | -5.79 | <.001 |  |  |
|  |  |  |  |  |  |  |  |  |
| WSAS | Intercept | 10.80 | 3.24 | 128 | 3.33 | 0.001 | 842.54 | 4.98 |
|  | First treatment | 0.74 | 0.08 | 128 | 9.59 | <.001 |  | Residual variance: 1.87 |
|  | Helpfulness | -0.04 | 1.43 | 128 | -0.02 | 0.98 |  |  |
|  | Improvement | -3.51 | 0.74 | 128 | -4.74 | <.001 |  |  |

**Table S5**

This table presents the findings of ANOVAs comparing the mixed models with random effects to the equivalent linear models without random effects, to evaluate whether the variance components of the random effects are significantly greater than zero.

| Outcome  Variable | Model type | *df* | AIC | BIC | Log-likelihood | L-ratio | *p*-value |
| --- | --- | --- | --- | --- | --- | --- | --- |
| GAD-7 | Mixed Model | 9 | 746.82 | 772.49 | -364.41 | 3.41e^-13^ | 1.00 |
|  | Linear Model | 8 | 744.82 | \|  \| \| --- \|   767.64 |  |  |  |
|  |  |  |  |  |  |  |  |
| PHQ-9 | Mixed Model | 9 | 741.91 | 767.58 | -361.96 | 3.41e^-13^ | 1.00 |
|  | Linear Model | 8 | 739.91 | 762.73 |  |  |  |
|  |  |  |  |  |  |  |  |
| WSAS | Mixed Model | 9 | 842.54 | 868.20 | -412.27 | 1.14e^-13^ | 1.00 |
|  | Linear Model | 8 | 840.54 | 863.35 |  |  |  |

*Note*. The linear mixed models might not provide a statistically better fit due to their greater number of parameters; the inclusion of a random effect uses an additional degree of freedom compared to linear models, and the sample size is relatively small.

**Table S6**

|  | | Linear Regressions | | | | | | | |
| --- | --- | --- | --- | --- | --- | --- | --- | --- | --- |
| OV | Regression Type | | EV | *β* | *SE* | *t*-value | | *p*-value* | Model Adj. R^2^ |
| Δ GAD-7 | Univariable | | Helpfulness | -2.43 | 0.98 | -2.48 | | 0.01 | 0.08 |
|  | Univariable | | Improvement | -2.35 | 0.48 | -4.91 | | < .001 | 0.19 |
|  | Multivariable | | Helpfulness & Improvement | 0.49 | 1.16 | 0.42 | | 1.00 | 0.19 |
|  |  |  |  | -2.50 | 0.60 | -4.15 | | < .001 |  |
|  |  | |  |  |  |  | |  |  |
| Δ PHQ-9 | Univariable | | Helpfulness | -1.87 | 0.97 | -1.93 | | 0.06 | 0.06 |
|  | Univariable | | Improvement | -2.30 | 0.47 | -4.92 | | < .001 | 0.18 |
|  | Multivariable | | Helpfulness & Improvement | 1.29 | 1.13 | 1.14 | | 0.78 | 0.18 |
|  |  |  |  | -2.70 | 0.59 | -4.61 | | < .001 |  |
|  |  | |  |  |  |  | |  |  |
| Δ WSAS | Univariable | | Helpfulness | -3.22 | 1.25 | -2.58 | | 0.01 | 0.06 |
|  | Univariable | | Improvement | -3.07 | 0.61 | -5.06 | | < .001 | 0.17 |
|  | Multivariable | | Helpfulness & Improvement | 0.58 | 1.48 | 0.40 | | 1.00 | 0.17 |
|  |  |  |  | -3.26 | 0.77 | -4.26 | | < .001 |  |
|  |  | |  |  |  |  |  | |  |
|  | | Logistic Regressions | | | | | | | |
| OV | Regression Type | | EV | *OR* | *SE* | *z*-value | *p*-value* | | AIC |
| Recovery | Univariable | | Helpfulness | 6.92 | 0.52 | 3.73 | < .001 | | 158.55 |
|  | Univariable | | Improvement | 3.85 | 0.32 | 4.27 | < .001 | | 152.49 |
|  | Multivariable | | Helpfulness & Improvement | 2.36 | 0.63 | 1.38 | 0.17 | | 150.59 |
|  |  |  |  | 2.89 | 0.37 | 2.91 | 0.01 | |  |
|  |  | |  |  |  |  |  | |  |
| Reliable Impro-vement | Univariable | | Helpfulness | 3.29 | 0.45 | 2.60 | 0.01 | | 182.25 |
|  | Univariable | | Improvement | 4.22 | 0.31 | 4.59 | < .001 | | 160.53 |
|  | Multivariable | | Helpfulness & Improvement | 0.61 | 0.61 | -0.83 | 0.41 | | 162.82 |
|  |  |  |  | 5.16 | 0.41 | 4.00 | < .001 | |  |
|  |  | |  |  |  |  |  | |  |
| Reliable Recovery | Univariable | | Helpfulness | 5.16 | 0.53 | 3.10 | 0.002 | | 163.26 |
|  | Univariable | | Improvement | 4.66 | 0.34 | 4.50 | < .001 | | 145.81 |
|  | Multivariable | | Helpfulness & Improvement | 1.15 | 0.66 | 0.22 | 0.83 | | 147.76 |
|  |  |  |  | 4.44 | 0.41 | 3.65 | < .001 | |  |

*Outputs and model summaries for linear and logistic regressions of questionnaire-based clinical outcomes and patient-rated treatment helpfulness and improvement.*

*Note.* Number of treatment sessions, gender, and age were included as covariates in all models. The adjusted R^2^ reflects the gain in variance explained by the explanatory variables, over that explained by the covariates. EV = Explanatory Variable, OV = Outcome Variable, SE = standard error, AIC = Akaike’s Information Criterion. Δ GAD-7 (PHQ-9, WSAS) = change scores of the Generalised Anxiety Disorder 7-item Scale (Patient Health Questionnaire 9-Item Scale, Work and Social Adjustment Scale); *reported *p*-values are the values obtained after applying the Holm-Bonferroni adjustment as described in the methods.

**Table S7**

*Means (standard deviations) of the continuous outcome variables per category of NHS Talking Therapies treatment outcomes.*

|  |  | **NHS Talking Therapies Treatment Outcome** | | | | | | | |
| --- | --- | --- | --- | --- | --- | --- | --- | --- | --- |
|  |  | Recovery | |  | Reliable Improvement | |  | Reliable Recovery | |
|  |  | Yes | No |  | Yes | No |  | Yes | No |
|  |  |  |  |  |  |  |  |  |  |
| Number of Participants | | 64 | 60 |  | 77 | 58 |  | 54 | 70 |
|  |  |  |  |  |  |  |  |  |  |
| **GAD-7** | First treatment | 11.72 (3.92) | 14.13 (3.77) |  | 13.53 (4.05) | 10.67 (4.20) |  | 12.54 (3.69) | 13.16 (4.26) |
|  | Last treatment | 4.80 (1.66) | 12.13 (4.19) |  | 6.18 (3.46) | 10.53 (5.18) |  | 4.61 (1.69) | 11.23 (4.49) |
|  | Δ | -6.92 (4.23) | -2.00 (4.22) |  | -7.35 (3.55) | -0.14 (2.78) |  | -7.93 (3.82) | -1.93 (3.92) |
|  |  |  |  |  |  |  |  |  |  |
|  | First treatment | 11.66 (4.40) | 14.57 (4.10) |  | 12.91 (4.98) | 12.16 (4.11) |  | 11.91 (4.60) | 13.96 (4.22) |
| **PHQ-9** | Last treatment | 5.75 (2.36) | 13.23 (3.92) |  | 7.08 (3.78) | 11.66 (5.11) |  | 5.50 (2.27) | 12.36 (4.32) |
|  | Δ | -5.91 (4.19) | -1.33 (4.30) |  | -5.83 (4.28) | -0.50 (3.27) |  | -6.41 (4.33) | -1.60 (4.08) |
|  |  |  |  |  |  |  |  |  |  |
| **WSAS** | First treatment | 18.08 (5.14) | 22.63 (5.90) |  | 19.56 (6.03) | 19.91 (6.42) |  | 18.06 (5.22) | 22.00 (5.95) |
|  | Last treatment | 11.19 (5.68) | 21.38 (6.27) |  | 13.18 (7.27) | 18.88 (7.60) |  | 10.69 (5.56) | 20.31 (6.70) |
|  | Δ | -6.89 (6.03) | -1.25 (4.96) |  | -6.38 (5.77) | -1.03 (5.01) |  | -7.38 (6.15) | -1.69 (5.03) |

*Note.* NHS = National Health Service; GAD-7 = Generalised Anxiety Disorder 7-item Scale; PHQ-9 = Patient Health Questionnaire 9-Item Scale; WSAS = Work and Social Adjustment Scale; Δ = change score.

**Table S8**

| Change Score | NHS Treatment Outcome | | Mean Difference | | | | | |  | Independent Samples *t*-test | | | | |
| --- | --- | --- | --- | --- | --- | --- | --- | --- | --- | --- | --- | --- | --- | --- |
|  |  |  | 95% CI | | | | | *d* |  | *t* | | *df* | | *p* |
| Δ GAD-7 | Recovery | | [0.78, 1.54] | | | | | 1.16 |  | 6.48 | | 122 | | <.001 |
|  | Reliable Improvement | | [1.79, 2.66] | | | | | 2.23 |  | 12.80 | | 133 | | <.001 |
|  | Reliable Recovery | | [1.14, 1.95] | | | | | 1.55 |  | 9.22 | | 122 | | <.001 |
|  |  | | | |  |  | |  |  |  | |  | |  |
| Δ PHQ-9 | Recovery | | [0.70, 1.69] | | | | | 1.08 |  | 5.99 | | 122 | | <.001 |
|  | Reliable Improvement | | [0.99, 1.75] | | | | | 1.37 |  | 7.90 | | 133 | | <.001 |
|  | Reliable Recovery | | [0.76, 1.53] | | | | | 1.15 |  | 6.33 | | 122 | | <.001 |
|  |  | | | |  |  | |  |  |  | |  | |  |
| Δ WSAS | Recovery | | [0.64, 1.39] | | | | | 1.02 |  | 5.67 | | 122 | | <.001 |
|  | Reliable Improvement | | [0.62, 1.34] | | | | | 0.98 |  | 5.64 | | 133 | | <.001 |
|  | Reliable Recovery | | [0.65, 1.40] | | | | | 1.03 |  | 5.67 | | 122 | | <.001 |
|  | |  | |  | | |  |  |  | |  | |  |  |

*Results of independent-samples t-tests of differences on change scores in clinical questionnaires between participants who met and did not meet criteria for each NHS Talking Therapies category.*

*Note.* Effect sizes were computed using pooled standard deviations.

**Figure S3**

*Probability of Meeting the Criteria of NHS Talking Therapies Treatment Outcomes by Categories of Helpfulness and Improvement.*


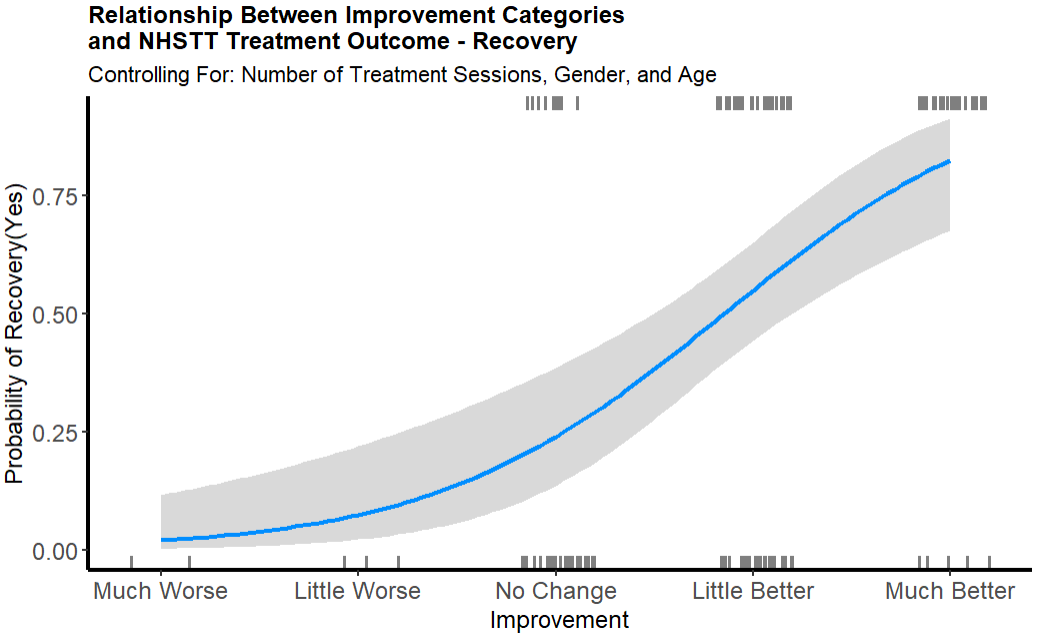

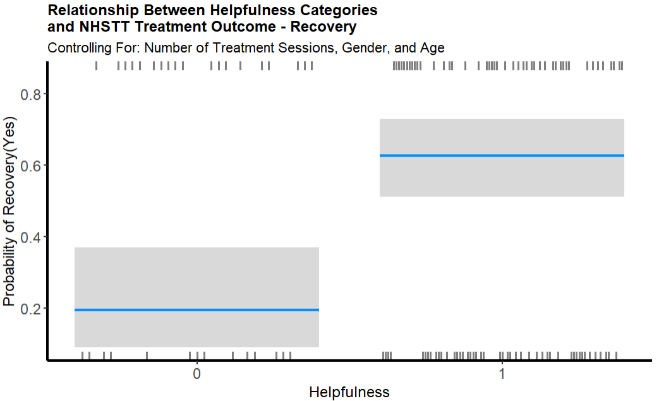


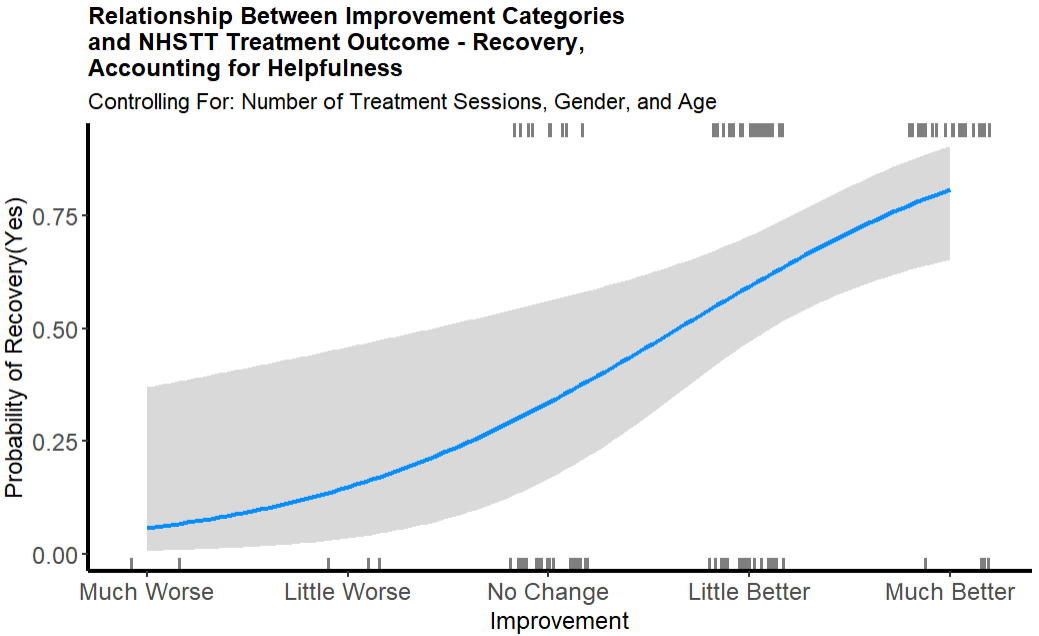

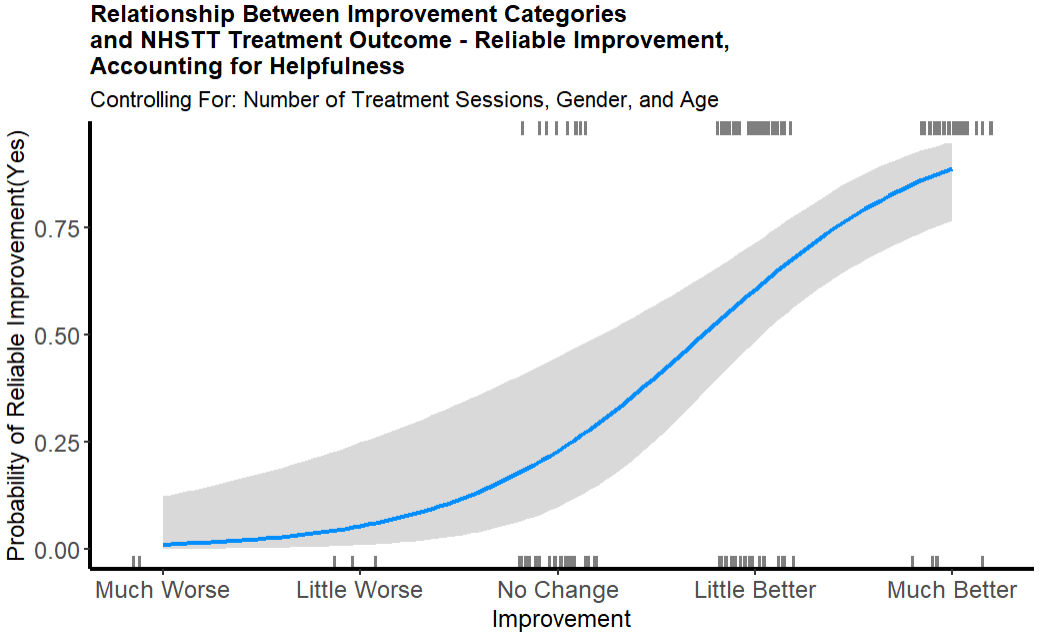

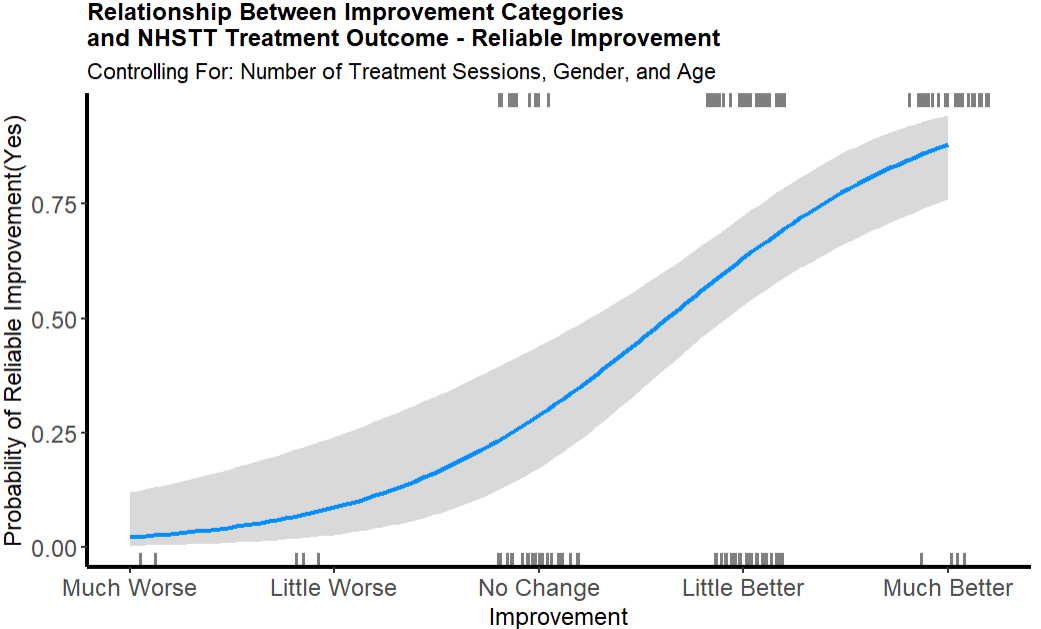

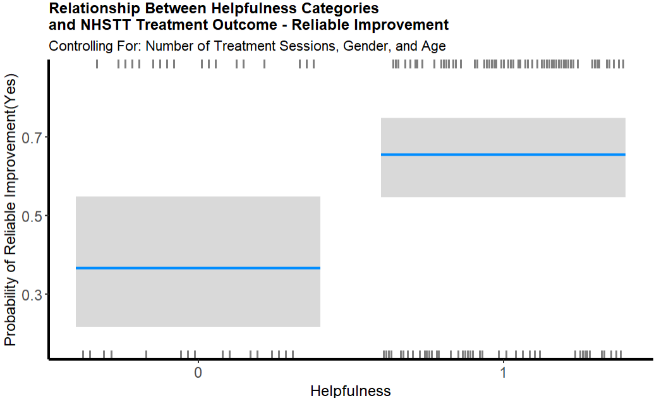


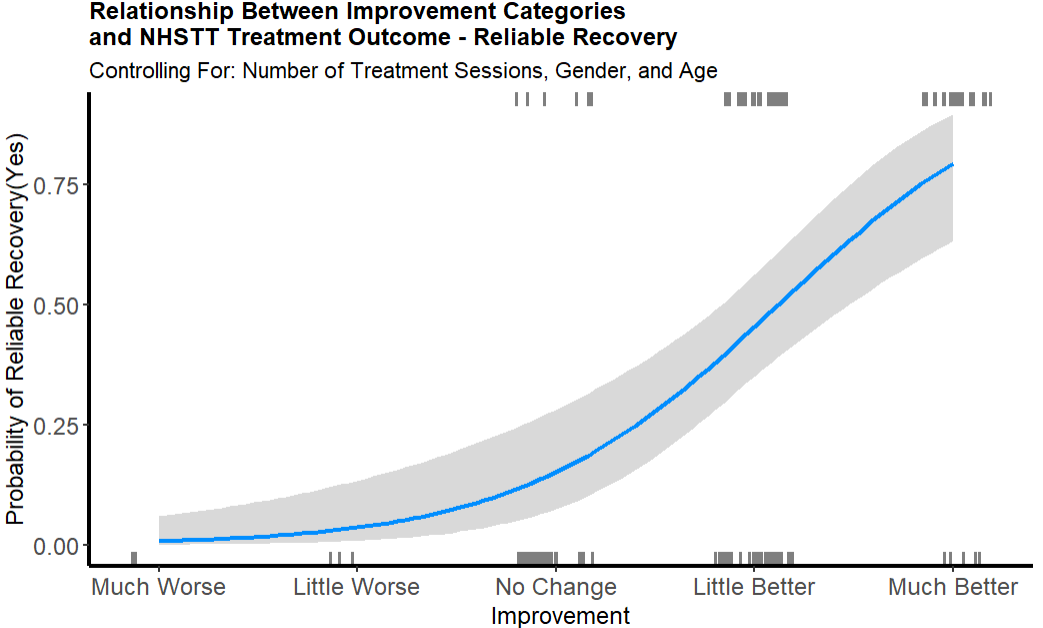

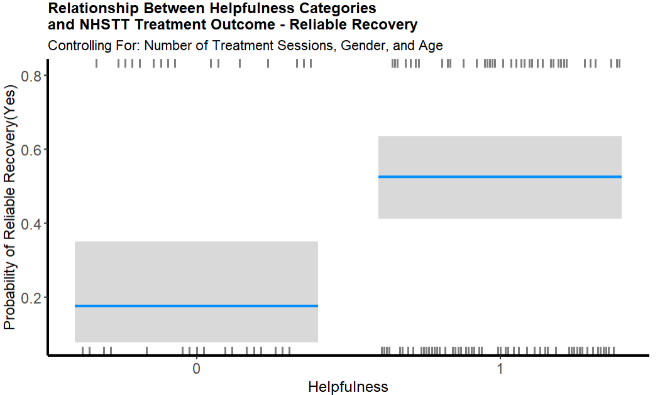


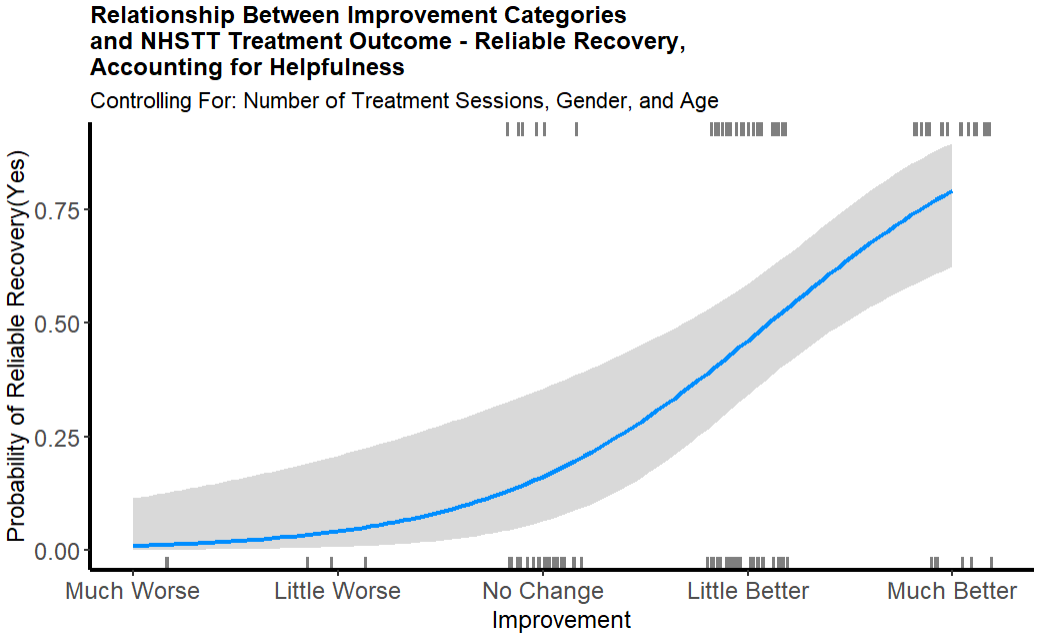


*Note*. For Helpfulness – 0 = “no”, 1 = ”yes”.

**References**

Catarino, A., Bateup, S., Tablan, V., Innes, K., Freer, S., Richards, A., Stott, R., Hollon, S. D., Chamberlain, S. R., Hayes, A., &amp; Blackwell, A. D. (2018). Demographic and clinical predictors of response to internet-enabled cognitive–behavioural therapy for depression and anxiety. BJPsych Open, 4(5), 411–418. https://doi.org/10.1192/bjo.2018.57

Davies, M. R., Kalsi, G., Armour, C., Jones, I. R., McIntosh, A. M., Smith, D. J., Walters, J. T., Bradley, J. R., Kingston, N., Ashford, S., Beange, I., Brailean, A., Cleare, A. J., Coleman, J. R., Curtis, C. J., Curzons, S. C., Davis, K. A., Dowey, L. R. C., Gault, V. A., . . . Breen, G. (2019). The Genetic Links to Anxiety and Depression (GLAD) Study: Online recruitment into the largest recontactable study of depression and anxiety. Behaviour Research and Therapy, 123, 103503. https://doi.org/10.1016/j.brat.2019.103503

Harris, P. A., Taylor, R., Minor, B. L., Elliott, V., Fernandez, M., O’Neal, L., McLeod, L., Delacqua, G., Delacqua, F., Kirby, J., &amp; Duda, S. N. (2019). The REDCap consortium: Building an international community of software platform partners. Journal of Biomedical Informatics, 95, 103208. <https://doi.org/10.1016/j.jbi.2019.103208>

McGregor, T., Carr, E., Barry, T., Catarino, A., Craske, M. G., Davies, M. R., ... & Eley, T. C. (2025). Self-report measures of fear learning and extinction and their association with internet-based cognitive behavioural therapy outcome. Behaviour Research and Therapy, 187, 104705. https://doi.org/10.1016/j.brat.2025.104705

Shrestha, N. (2020). Detecting multicollinearity in regression analysis. American Journal of Applied Mathematics and Statistics, 8(2), 39–42. <https://doi.org/10.12691/ajams-8-2-1>
